# Essential role of vesicular nucleotide transporter in vesicular storage and release of nucleotides in platelets

**DOI:** 10.14814/phy2.12034

**Published:** 2014-06-11

**Authors:** Miki Hiasa, Natsuko Togawa, Takaaki Miyaji, Hiroshi Omote, Akitsugu Yamamoto, Yoshinori Moriyama

**Affiliations:** 1 Department of Membrane Biochemistry, Okayama University Graduate School of Medicine, Dentistry and Pharmaceutical Sciences, Okayama, Japan; 2 Advanced Science Research Center, Okayama University, Okayama, Japan; 3 Department of Cell Biology, Nagahama Institute of Technology, Nagahama, Japan

**Keywords:** ADP, ATP, platelets, SLC17A9, vesicular nucleotide transporter

## Abstract

Nucleotides are stored in the dense granules of platelets. The release of nucleotides triggers one of the first steps in a series of cascades responsible for blood coagulation. However, the mechanism of how the nucleotides are accumulated in the granules is still far less understood. The transporter protein responsible for storage of nucleotides in the neuroendocrine cells has been identified and characterized. We hypothesized that the vesicular nucleotide transporter (VNUT) is also involved in the vesicular storage of nucleotides in platelets. In this article, we present three lines of evidence that VNUT is responsible for the vesicular storage of nucleotides in platelets and that vesicular ATP transport is crucial for platelet function, detection and characterization of VNUT activity in platelets isolated from healthy humans and MEG‐01 cells, RNA interference experiments on MEG‐01 cells, and studies on nucleotide transport and release with a selective inhibitor.

## Abstract (precis)

VNUT is highly expressed and associated with dense granules in platelets. VNUT plays an essential role in vesicular storage of nucleotide in platelets.

## Introduction

Extracellular nucleotides function as intercellular messengers and cause various physiological or pathological responses upon binding to purinoceptors on the target cells (Burnstock 2007). Platelets play an essential role in hemostasis and thrombosis and are released by the pro‐platelets of megakaryocytes (Malmgren 1986; Siess 1989; Lemons et al. 1997; Vischer and Wollheim 1998; McNicl and Israels 1999). Upon stimulation, platelets secrete ATP and ADP, which facilitate platelet aggregation through distinct signaling pathways: ATP binds to the P2X_1_ receptor and initiates rapid Ca^2+^ influx followed by platelet shape change, leading to platelet activation induced by low concentrations of collagen (Clifford et al. 1998; Gachet 2006). ADP binds to the P2Y_1_ receptor, causing intracellular Ca^2+^ mobilization, shape change, and initiation of aggregation (Fabre et al. 1999; Gachet 2006). ADP also binds to P2Y_12_ receptor and the resultant decrease in cAMP potentiates the aggregation and secretion induced by all known platelet agonists such as thrombin, collagen, and serotonin (Gachet 2006). Thus, purinergic chemical transmission plays a crucial role in platelet activation and their receptors are potential targets for antithrombotic drugs (Hollopeter et al. 2001).

In spite that the function of purinoceptors in platelets is understood well, the mechanism of how platelets store nucleotides is far less understood. Platelets contain two types of secretory granules, the *α* granules and dense granules (Da Prada et al. 1982; Njus et al. 1986; McNicl and Israels 1999). While *α* granules contain mainly polypeptides such as fibrinogen, von Willebrand factor, growth factor, and protease inhibitors, dense granules contain a high concentration of ATP and ADP (up to ~0.7 mol/L) as well as serotonin and divalent cations (Da Prada et al. 1982; Njus et al. 1986; McNicl and Israels 1999). It has been shown that dense granules contain a vacuolar type proton pump that acidifies the interior of the granule (Dean et al. 1984). Serotonin is actively transported into the granules through vesicular monoamine transporter coupled with the *Δ*pH established by the proton pump (Wilkins et al. 1978; Carty et al. 1981). In analogy to serotonin transport, it has been speculated that secondary active transporter is involved in nucleotide storage in the granules; however, studies to demonstrate this possibility were all unsuccessful. An alternative hypothesis is that the multidrug resistant associated protein 4 (MRP4/ABCC4), a member of ABC‐type drug transporter family, is involved in the vesicular storage of nucleotides in platelets since this protein transports various anionic compounds at the expense of ATP hydrolysis (Jedlitschky et al. 2004, 2012). MRP4 is present in dense granules and the platelet membrane vesicles transport cyclic GMP, cyclic AMP, and ADP upon incubation with ATP in a vanadate‐sensitive fashion (Jedlitschky et al. 2004, 2012). However, because MRP4 is polyspecific in nature and recognizes structurally unrelated compounds as substrates, it is difficult to explain how MRP4 can selectively accumulate ATP and ADP in the granules.

Vesicular nucleotide transporter (VNUT, SLC17A9 protein) is the latest member of SLC17 anion transporter family that localizes to various ATP storage organelles such as chromaffin granules and synaptic vesicles (Sawada et al. 2008; Larsson et al. 2012). VNUT actively transports various nucleotides using the membrane potential (*Δψ*; inside positive) that is established by vacuolar proton ATPase. The ATP uptake requires a low concentration of Cl^−^ (of about 4 mmol/L) and is inhibited by Evans blue, 4,4′‐diisothiocyanostilbene 2,2′‐disulfonic acid (DIDS), and keto acids such as acetoacetate, which are common inhibitors for the SLC17 transporter family (Sawada et al. 2008; Juge et al. 2010). ATP and ADP with chelated divalent cations are the preferred transport substrates of VNUT (Miyaji et al. 2011). Furthermore, very recent studies have shown that secretion of ATP from PC12 cells, purinergic neuronal cells, T cells, biliary epithelial cells, airway epithelial cells, macrophages, pancreatic *β* cells, and lung cancer cell line were impaired in VNUT knock down, indicating that VNUT is of primary importance for vesicular storage and release of nucleotides (Sawada et al. 2008; Tokunaga et al. 2010; Sathe et al. 2011; Larsson et al. 2012; Takai et al. 2012; Geisler et al. 2013; Sakaki et al. 2013; Sesma et al. 2013).

Therefore, in this study, we investigated the possible involvement of VNUT in the vesicular storage and release of nucleotides in platelets.

## Materials and Methods

### Preparations

Human platelets were obtained from volunteer healthy donors in compliance with the guidelines of the ethics committee of Okayama University, permission number 1388. The blood (36 mL) was mixed with 4 mL of 3.8% sodium citrate immediately after drawing and centrifuged at 230*g* for 15 min. The platelet‐rich plasma (supernatant) was centrifuged at 1200*g* for 6 min. The resultant supernatant was carefully discarded and the pellet (platelet fraction) was washed with 10 mmol/L MOPS‐Tris (pH 7.0) buffer containing 300 mmol/L sucrose, 5 mmol/L EDTA, 10 *μ*g/mL leupeptin, and 10 *μ*g/mL pepstatin A (SME buffer). After centrifugation at 1200*g* for 6 min, the pellet (platelets) was suspended in the same buffer and kept on ice until use.

MEG‐01 clonal megakaryoblastic cells kindly provided by T. Nishi (ISIR, Osaka University) were cultured in RPMI medium with 10% fetal bovine serum and 2 mg/mL sodium bicarbonate and then incubated at 37°C under 5% CO_2_.

Rabbit polyclonal antibodies for human VNUT, V‐ATPase, and *N*‐ethylmaleimide sensitive factor (NSF) were used (Moriyama et al. 1995; Sawada et al. 2008). Sheep polyclonal antibody for cellubrevin (Abcam, Cambridge, U.K.), goat polyclonal antibody for *p*‐selectin (Santa Cruz Biotechnology Inc., Santa Cruz, CA) and for MRP4 (Abnova, Taipei, Taiwan), mouse monoclonal antibody for GPIIb/GPIIIa (BMA Biomedicals, Augst, Switzerland), VAMP8 (Santa Cruz Biotechnology Inc.), LAMP1 (StressMarq Biosciences Inc., Victoria, BC, Canada), and rat monoclonal antibody for serotonin (Millipore, Billerica, MA) were purchased. Human VNUT protein was purified to homogeneity according to the published procedures (Leviatan et al. 2010).

### Immunofluorescence microscopy

Indirect immunofluorescence microscopy was performed as previously described. Platelets or MEG‐01 cells on poly‐l‐lysine‐coated coverslips were fixed with 0.4% paraformaldehyde in phosphate‐buffered saline (PBS) for 30 min. The first antibody treatment was performed with anti‐human VNUT antibody diluted 1:200 in PBS containing 0.5% bovine serum albumin (BSA) for 1 h at room temperature. Washing and secondary antibody treatment were performed according to the published procedures (Hayashi et al. 1999). The specimens were observed under an Olympus FV300 confocal laser microscope (Sawada et al. 2008).

### Immunoelectron microscopy

The pre‐embedding nanogold intensification method was used (Yamamoto and Masaki 2010). Pellet of platelets was fixed by 0.05% glutaraldehyde and 4% paraformaldehyde in 0.1 mol/L phosphate buffer (pH 7.4) for 60 min. The samples were then washed with PBS, and were frozen in liquid nitrogen after cryo‐protection in PBS containing 10%, 15%, and 20% sucrose successively. Cryosections of ~6 *μ*m were cut and attached to cover glass and processed for pre‐embedding nanogold gold intensification method (Yamamoto and Masaki 2010). Cryosections of the platelets were embedded in epoxy resin, and ultrathin sections were cut, and stained sequentially with uranyl acetate for 10 min and lead citrate for 1 min, respectively, and observed under a Hitachi H‐7600 electron microscope (Tokyo, Japan).

### Heat treatment and deglycosylation of platelets membrane proteins

Briefly, 40 *μ*g membrane fractions from human platelets were heat treated in 30 *μ*L aliquot containing 0.5% SDS (sodium dodecyl sulfate) and 40 mmol/L dithiothreitol at 75°C for 15 min. Then the samples were cooled to room temperature, then reacted with *N*‐glycosidase F (3000 units; New England Biolabs, Ipswich, MA), 1% NP‐40 for 1 h at 37°C according to the manufacturer's manual. Then, the samples were dissociated with SDS sample buffer and analyzed with polyacrylamide gel electrophoresis in the presence of SDS and the Western blotting.

### ATP uptake

The platelets or MEG‐01 cells were disrupted by nitrogen cavitation after equilibration at 1000 psi for 10 min and centrifuged at 1000*g* for 10 min to remove cell debris; the resultant supernatant was centrifuged at 160,000*g* for 2 h. The pellet (membrane vesicles) was suspended in SME buffer. The membrane vesicles (8.7 *μ*g protein per assay) were suspended in 120 *μ*L of 20 mmol/L MOPS‐Tris (pH 7.0), 300 mmol/L sucrose, 2 mmol/L magnesium acetate, 4 mmol/L KCl, 5 *μ*mol/L oligomycin, and 1 *μ*mol/L atractyloside and incubated in the absence or presence of 0.1 mmol/L glyoxylate (Sigma, St. Louis, MO) for 2 min at 30°C. Oligomycin was added to exclude any possible participation of ATP uptake by contaminating mitochondrial membrane (corresponded to <10% of total activity under the assay conditions employed). The assay was initiated by addition of 2 mmol/L [2,8‐^3^H] ATP (0.05 MBq/*μ*mol); 100 *μ*L aliquots were taken at the times indicated and filtered through 0.45 *μ*m nitrocellulose filters (Millipore). After washing with 6 mL cold buffer containing 20 mmol/L MOPS‐Tris (pH 7.0), 300 mmol/L sucrose, 2 mmol/L magnesium acetate, and 4 mmol/L KCl, the remaining radioactivity on the filters was counted in a liquid scintillation counter (PerkinElmer, Waltham, MA). More than 90% of the radioactive ATP was not degraded under the assay conditions employed. To evaluate background ATP binding to membrane, same assay was carried out under ice‐cold conditions.

### ATP and ADP release

MEG‐01 cells (2.0 × 10^5^ cells) were incubated in a Krebs–Ringer solution that comprised 128 mmol/L NaCl, 1.9 mmol/L KCl, 1.2 mmol/L KH_2_PO_4_, 1.3 mmol/L MgSO_4_, 26 mmol/L NaHCO_3_, 2.4 mmol/L CaCl_2_, 10 mmol/L d‐glucose, 10 mmol/L 4‐(2‐hydroxyethyl)‐1‐piperazineethanesulfonic acid (HEPES)‐Tris (pH 7.4), and 0.2% BSA in the absence or presence of serial dilution of glyoxylate for 30 min at 37°C. The cells were incubated under low Ca^2+^ (0.2 mmol/L CaCl_2_, 3.8 mmol/L MgSO_4_, 1 mmol/L EGTA) condition or stimulated by the addition of A23187 (Ca^2+^ ionophore) to a final concentration of 5 *μ*mol/L at 37°C. After aliquots were taken at 20 min, the amount of ATP was measured by bioluminescence method (Invitrogen, Carlsbad, CA) based on luciferin–luciferase reaction with BLR‐101C Luminescence Reader (Aloka, Tokyo, Japan).

For ADP conversion into ATP, the following reaction buffer was added to ADP in Krebs–Ringer solution: 40 mmol/L Tricine (pH 7.75), 40 mmol/L magnesium sulfate, 10 mmol/L KCl, 40 *μ*mol/L phosphoenolpyruvate (Sigma), and 40 U/mL pyruvate kinase (Oriental Yeast Co., Ltd., Tokyo, Japan) and incubated for 5 min at room temperature. The net amount of ADP was calculated by subtracting the control (no pyruvate kinase added) ATP value from the ATP value in the presence of pyruvate kinase.

### RNAi

Amaxa Cell Line Nucleofector Kit C (Lonza, Switzerland) was used for transfection of 600 nmol/L AllStars negative control siRNA (Qiagen, Hilden, Germany) or human *SLC17A9* siRNA (SI03172078; Qiagen). Ca^2+^ ionophore‐stimulated ATP secretion was assayed 48 h later as described (Sawada et al. 2008; Larsson et al. 2012).

### Real‐time PCR

RNA was purified from cultured MEG‐01 cells (8 × 10^6^ cells) using RNeasy kit (Qiagen) according to the manufacturer's instructions. Real‐time quantitative PCR was performed using SYBR Premix Ex Taq II (TAKARA BIO, Shiga, Japan) containing the double‐stranded DNA‐binding fluorescent probe SYBR Green and all necessary components except primers. Quantitative PCR conditions included an initial denaturation step of 95°C for 30 sec followed by 40 cycles of 95°C for 15 sec, and 60°C for 30 sec. Standards and samples were analyzed in triplicate. The following primers were used: human VNUT, TGGTCTTTGCATCAGCCTCCATCGG (forward), GTGTTGGCCACACCAAACAGAAAGC (reverse).

### Purification of vesicular neurotransmitter transporters

The cDNAs of rat VGLUT2, human VNUT, mouse VEAT, rat VGAT, rat VMAT2, and human VAChT have been described previously (Juge et al. 2010). These transporters were expressed in insect cells or *Escherichia coli*, purified and reconstituted into liposomes and assayed (Juge et al. 2010). In brief, insect cells (1–2 × 10^8^ cells) were suspended in buffer containing 20 mmol/L Tris‐HCl (pH 8.0), 0.1 mol/L sodium acetate, 10% glycerol, 0.5 mmol/L dithiothreitol, 10 *μ*g/mL pepstatin A, and 10 *μ*g/mL leupeptin and disrupted by sonication with a TOMY UD200 tip sonifier (TOMY, Tokyo, Japan). Cell lysates were centrifuged at 700*g* for 10 min to remove cell debris and the resultant supernatant was centrifuged at 160,000*g* for 1 h. The pellet (membrane fraction) was suspended in buffer containing 20 mmol/L MOPS‐Tris (pH 7.0), 10% glycerol, 10 *μ*g/mL pepstatin A, and 10 *μ*g/mL leupeptin at approximately 2 mg protein/mL. The membrane fraction was solubilized with 2% octylglucoside buffer containing 20 mmol/L MOPS‐Tris (pH7.0), 10% glycerol, 1 *μ*g/mL pepstatin A, and 1 *μ*g/mL leupeptin. After centrifugation at 260,000*g* for 30 min, the supernatant was added to 1 mL Ni‐NTA Superflow resin (Qiagen) and incubated for 4 h at 4°C. The resin was washed with 10 mL of 20 mmol/L MOPS‐Tris (pH 7.0), 5 mmol/L imidazole, 20% glycerol, and 1% octylglucoside in a column. The transporter was eluted from the resin with 3 mL of the same buffer containing 80 mmol/L imidazole. The eluate containing purified transporter was stored at −80°C where it was stable without loss of activity for at least a few months.

### Reconstitution of vesicular neurotransmitter transporters

Reconstitution of purified recombinant vesicular neurotransmitter transporters into liposomes was carried out by the freeze‐thaw method described elsewhere (Juge et al. 2010). Briefly, 10–20 *μ*g vesicular neurotransmitter transporter was mixed with 500‐*μ*g liposomes, frozen at −80°C, and left at this temperature for 15 min. The mixture was thawed quickly by holding the sample tube in the hand and diluted 60‐fold with reconstitution buffer (20 mmol/L MOPS‐Tris [pH 7.0], 0.5 mmol/L dithiothreitol, 0.15 mol/L sodium acetate, and 5 mmol/L magnesium acetate). The buffer composition was changed as necessary. Reconstituted proteoliposomes were pelleted by centrifugation at 200,000*g* for 1 h at 4°C and suspended in 0.2 mL of 20 mmol/L MOPS‐Tris (pH 7.0), containing 0.15 mol/L sodium acetate and 5 mmol/L magnesium acetate. Asolectin liposomes were prepared as follows. Soybean lecithin (20 mg; Sigma type IIS) was suspended in 2 mL of 20 mmol/L MOPS‐NaOH (pH 7.0) containing 1 mmol/L dithiothreitol. The mixture was sonicated in a bath‐type sonicator until clear, divided into small aliquots, and stored at −80°C until use.

In some experiments, purified VMAT2 or VAChT were co‐reconstituted with bacterial F‐ATPase into liposomes by the freeze‐thaw method described elsewhere (Juge et al. 2010). In brief, 20 *μ*g VMAT2 or VAChT was mixed with 90 *μ*g F‐ATPase and liposomes (0.5 mg lipid), frozen at −80°C, and left at this temperature for at least 15 min. The mixture was thawed quickly by holding the sample tube in the hand and diluted 60‐fold with 20 mmol/L MOPS‐Tris (pH 7.0), 0.1 mol/L potassium acetate, 5 mmol/L magnesium acetate, and 0.5 mmol/L dithiothreitol. The buffer composition was changed as necessary. Reconstituted proteoliposomes were pelleted by centrifugation at 200,000*g* for 1 h at 4°C and suspended in 0.4 mL of 20 mmol/L MOPS‐Tris (pH 7.0) containing 0.1 mol/L potassium acetate and 5 mmol/L magnesium acetate and used for experiment within a day of preparation.

### Uptake of neurotransmitters

Assays were carried out by the gel‐permeation procedure as described previously (Juge et al. 2010). For valinomycin‐evoked uptake, a 500 *μ*L aliquot of reaction mixture containing 20 mmol/L MOPS‐Tris (pH 7.0), 5 mmol/L magnesium acetate, 10 mmol/L KCl, 0.14 mol/L potassium acetate, 2 *μ*mol/L valinomycin, and 100 *μ*mol/L [2,8‐^3^H] ATP (0.5 MBq/*μ*mol; PerkinElmer) or 100 *μ*mol/L [2,3‐^3^H] l‐glutamate (0.5 MBq/*μ*mol; PerkinElmer) or 100 *μ*mol/L [2,3‐^3^H] l‐aspartate (0.5 MBq/*μ*mol; PerkinElmer) or [2,3‐^3^H] GABA (0.5 MBq/*μ*mol; PerkinElmer) was incubated for 3 min at 27°C. Proteoliposomes (1.0 *μ*g protein per assay) were added to the mixture to initiate the reaction and incubated for a further 1 min. Aliquots (130 *μ*L) were taken at the times indicated and centrifuged through a Sephadex G‐50 (fine) spin column at 760*g* for 2 min. Radioactivity in the eluate was counted by liquid scintillation counter.

For ATP‐driven transport, proteoliposomes containing both VMAT2 or VAChT and F‐ATPase (5 *μ*g total protein each) were suspended in 20 mmol/L MOPS‐Tris (pH 7.0), 5 mmol/L magnesium acetate, 10 mmol/L KCl, and 0.1 mol/L potassium acetate and incubated for 3 min at 27°C. ATP at 5 mmol/L was added and the mixture was incubated for a further 1 min. The assay was initiated by addition of 10 *μ*mol/L [2‐^3^H] serotonin (0.5 MBq/*μ*mol; PerkinElmer) or 10 *μ*mol/L [methyl‐^3^H] acetylcholine (0.5 MBq/*μ*mol; American Radiolabeled Chemicals, St. Louis, MO). Aliquots (130 *μ*L) were taken at the times indicated and centrifuged through a Sephadex G‐50 (fine) spin column at 760*g* for 2 min. Radioactivity and protein concentration of the eluate were measured (PerkinElmer).

### Other procedures

Polyacrylamide gel electrophoresis in the presence of SDS and Western blotting were performed as described (Hayashi et al. 1999). Protein concentration was assayed using BSA as a standard (Schaffner and Weissmann 1973).

### Data analysis

If otherwise specified, numerical values are shown as the mean ± SEM (*n* = 3–6). Statistical significance was determined by Student's *t*‐test: **P < 0.01, ***P < 0.001.

## Results

### Dense granules in human platelets contain functional VNUT

**Figure 1.**
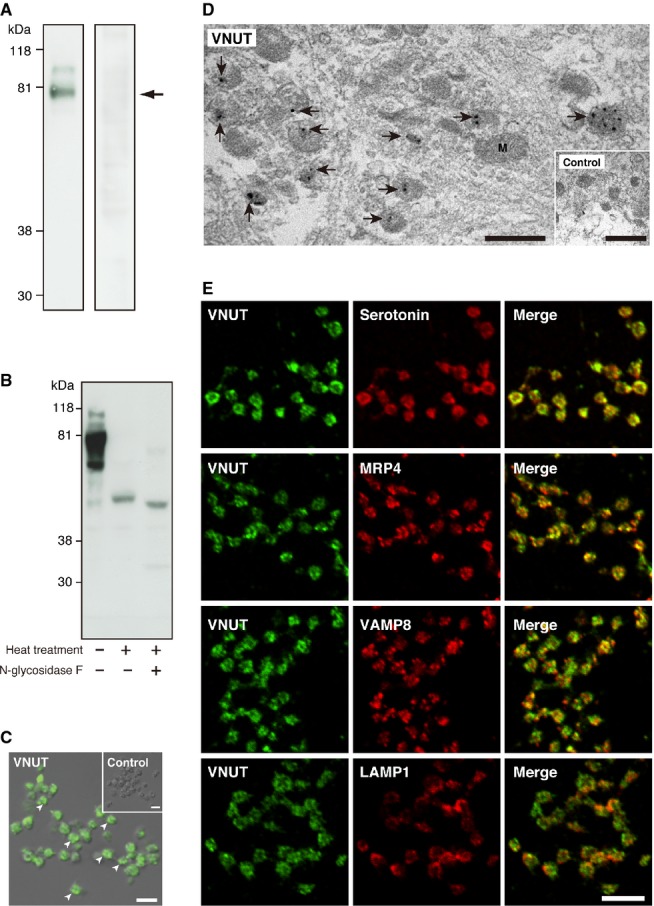
Vesicular nucleotide transporter (VNUT) was present in the dense granules of human platelets. (A) The membrane fraction from human platelets (20 *μ*g) was electrophoresed on a 10% polyacrylamide gel, transferred to a nitrocellulose membrane, and then subjected to Western blot analysis with anti‐hVNUT antibodies. The preabsorbed antibodies did not bind to the protein (right lane). The position of VNUT is marked by an arrow. (B) The membrane fraction from human platelets (30 *μ*g) was heat treated and then incubated at 37°C in the presence or absence of *N*‐glycosidase F as described in the Materials and Methods. Samples were then subjected to Western blot analysis with anti‐hVNUT antibodies. (C) Indirect immunofluorescence microscopy revealed that VNUT was expressed in platelets. (*Inset*) Shown is control staining with normal serum. Pictures merged with Nomarski images are shown. Typical punctate localizations are marked by arrowheads (scale bars: 5 *μ*m). (D) Immunoelectron microscopy revealed that VNUT localized to granules in platelets (Arrows). (*Inset*) Shown is background labeling with control serum. M, mitochondria (scale bars: 500 nm) (E) Human platelets were double‐immunostained with antibodies against VNUT (green) and markers (red): serotonin, MRP4, VAMP8, and LAMP1. Merged images are also shown (scale bar: 5 *μ*m)

As the first step of the study, we prepared platelets from the fresh blood of volunteers and examined whether VNUT exists in platelets by immunological techniques. Immunoblotting with purified anti‐VNUT antibodies identified an immunologically reactive protein with ~80 kDa in the platelets membranes (Fig. 1A, left lane). The immunoreactivity was not observed when the antibody was preabsorbed with purified VNUT protein (Fig. 1A, right lane). Apparent molecular weight of VNUT in platelets was higher than that of authentic VNUT protein (Sawada et al. 2008). We, therefore, denatured platelet membrane proteins by mild heat treatment at 75°C for 15 min in the presence of SDS, and then polyacrylamide gel electrophoresis was performed. As a result, immunoreactivity of VNUT migrated faster to the position of 55 kDa (Fig. 1B). Furthermore, apparent molecular weight of VNUT shifted from 55 to 53 kDa upon *N*‐glycosidase F treatment (Fig. 1B). Indirect immunofluorescence microscopy indicated that VNUT immunoreactivity is present in almost all platelets examined and exhibited a punctate distribution, while control IgG exhibited only background level staining (Fig. 1C). Then, we examined whether VNUT immunoreactivity was associated with granular components by immunoelectron microscopy. It was found that immunogold particles for VNUT were specifically associated with granular structure (Fig. 1D). Furthermore, double labeling immunofluorescence microscopy revealed that VNUT immunoreactivity was colocalized with serotonin, a neurotransmitter stored in the dense granules (Fig. 1E). VNUT was also colocalized with MRP4, an ATP‐driven polyspecific anion transporter protein, and localized in the dense granules, confirming the previous study (Fig. 1E) (Jedlitschky et al. 2004). VNUT was roughly colocalized with VAMP8, a vesicular‐associated SNARE protein, but not with a lysosome marker protein, LAMP1 (Fig. 1E). Taken together, it is suggested that VNUT is associated with serotonin‐containing dense granules (Da Prada et al. 1982).

ATP uptake by the membrane fraction upon incubation in buffer containing radiolabeled ATP and Mg^2+^ was assessed. Oligomycin was always included in the assay medium to reduce ATP hydrolysis and transport by mitochondrial membrane that may be present in the preparation. Under the assay conditions, the vesicles took up radiolabeled ATP (Table 1). Saturated binding of ATP at 2 mmol/L was 2.1 ± 0.9 nmol/mg protein that is 31% of total ATP uptake, indicating minor contribution of ATP binding. The ATP uptake was sensitive to bafilomycin A1, a V‐ATPase inhibitor indicating that an electrochemical gradient of protons across the membrane by vacuolar ATPase was essential for ATP uptake (Table 1). The ATP uptake was sensitive to Evans blue and DIDS, which are inhibitors of VNUT and stimulated by 4 mmol/L Cl^−^ (Table 1) (Bowman et al. 1988; Sawada et al. 2008). *Cis*‐inhibition experiments indicated that excess amounts of cold ATP and ADP strongly inhibited radiolabeled ATP uptake, whereas AMP was less effective (Table 1). AMP–PNP, a nonhydrolysable ATP analog and inorganic phosphate moderately inhibited the uptake (Table 1). These properties of ATP uptake by the platelet membrane vesicles were similar to those of ATP uptake in adrenal chromaffin granules and liposomes containing purified VNUT (Sawada et al. 2008) and suggested that functional VNUT is associated with platelet dense granules.

**Table 1.** Characterization of ATP uptake by platelets membrane fraction

	Compounds (*μ*mol/L)		Uptake, % of control
Inhibitors	Control		100.0 ± 3.0
	Evans Blue	10	57.9 ± 5.7
	DIDS	10	29.4 ± 10.9
	Bafilomycin A1	0.05	35.8 ± 3.1
Cl^−^ dependency	Control	4000	100.0 ± 19.7
		0	56.6 ± 22.2
*cis*‐inhibition	Control		100.0 ± 5.2
	ATP	5000	23.3 ± 0.3
	ADP	5000	3.6 ± 2.3
	AMP	5000	66.4 ± 2.5
	AMP‐PNP	5000	46.0 ± 5.0
	KH_2_PO_4_	5000	45.3 ± 3.3

The effect of inhibitors, Cl^−^, and nucleotides on ATP uptake by platelets membrane fraction after 5 min. Control activity (100%) corresponds to 6.7 ± 0.3 nmol/mg protein. For Cl^−^ dependence, an equivalent concentration of the potassium acetate in the reaction mixture was replaced with the indicated concentration of KCl. Data are mean ± SD, *n* = 3.

### Expression of functional VNUT in a human megakaryoblastic cells line

**Figure 2.**
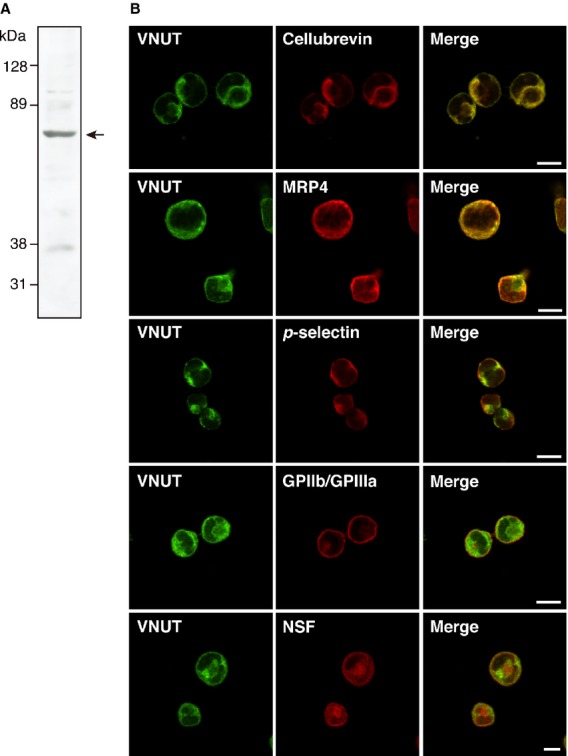
Expression of vesicular nucleotide transporter (VNUT) in MEG‐01 cells. (A) The membrane from MEG‐01 cells (20 *μ*g) was analyzed by Western blot with anti‐hVNUT antibodies. The position of VNUT is marked by an arrow. The positions of molecular markers were also indicated. (B) Double‐labeling immunohistochemistry indicated the localization of VNUT and the marker proteins cellubrevin, MRP4,* p*‐selectin, GPllb/GPllla, and NSF in MEG‐01 cells. Bars represent 10 *μ*m.

It is important to ask whether VNUT gene expression is correlated with vesicular storage and release of ATP. However, in platelets, this question cannot be answered since platelets lack nuclei and, thus, cannot be genetically manipulated. To assess the involvement of VNUT in vesicular storage and exocytosis of ATP in platelets, we used MEG‐01 cells, which are derived from human megakaryoblastic leukemia and can be induced to produce platelets (Ogura et al. 1985; Léon et al. 1997). It was found that MEG‐01 cells contained an immunological counterpart of VNUT with apparent molecular mass of 62 kDa by Western blotting (Fig. 2A). Double labeling immunofluorescence microscopy indicated that the VNUT immunoreactivity is colocalized with cellubrevin and MRP4 proteins, markers of secretory and dense granules, respectively (Fig. 2B) (Bernstein and Whiteheart 1999; Ambrosio et al. 2012). In contrast, the fluorescence of the VNUT antibody corresponded to a lesser extent to that of *p*‐selectin, a marker of *α* granules, GP IIb/GP IIIa, a marker of plasma membranes and cytosol, and a cytosolic protein, NSF (Fig. 2B).

**Figure 3.**
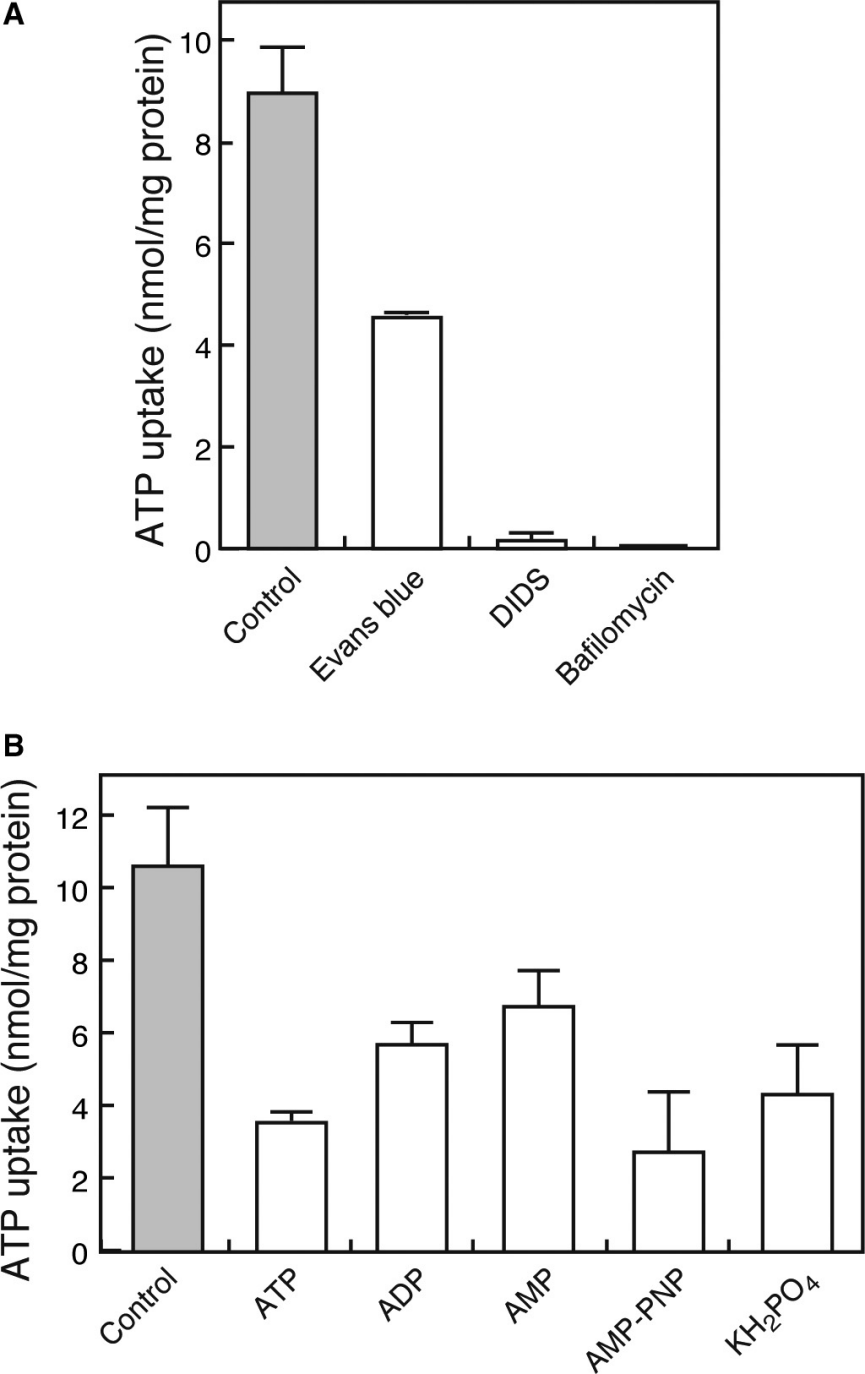
ATP transport by membrane vesicles in MEG‐01 cells. (A) ATP uptake by membrane vesicles isolated from MEG‐01 cells was measured after 5 min incubation in the presence of various ligands as follows: Evans blue at 10 *μ*mol/L, DIDS at 10 *μ*mol/L, or bafilomycin A1 at 50 nmol/L. (B) ATP uptake was measured in the absence or presence of various nucleotides at 5 min. Listed nucleotide derivatives were tested at 5 mmol/L.

We also prepared membrane vesicles from MEG‐01 cells and assayed ATP uptake. As shown in Figure 3, ATP uptake by these membrane vesicles also exhibited a pattern of inhibitor sensitivities similar to those of human platelets, suggesting that the VNUT expressed in MEG‐01 cells is functional.

### VNUT is responsible for nucleotide release from MEG‐01 cells

**Figure 4.**
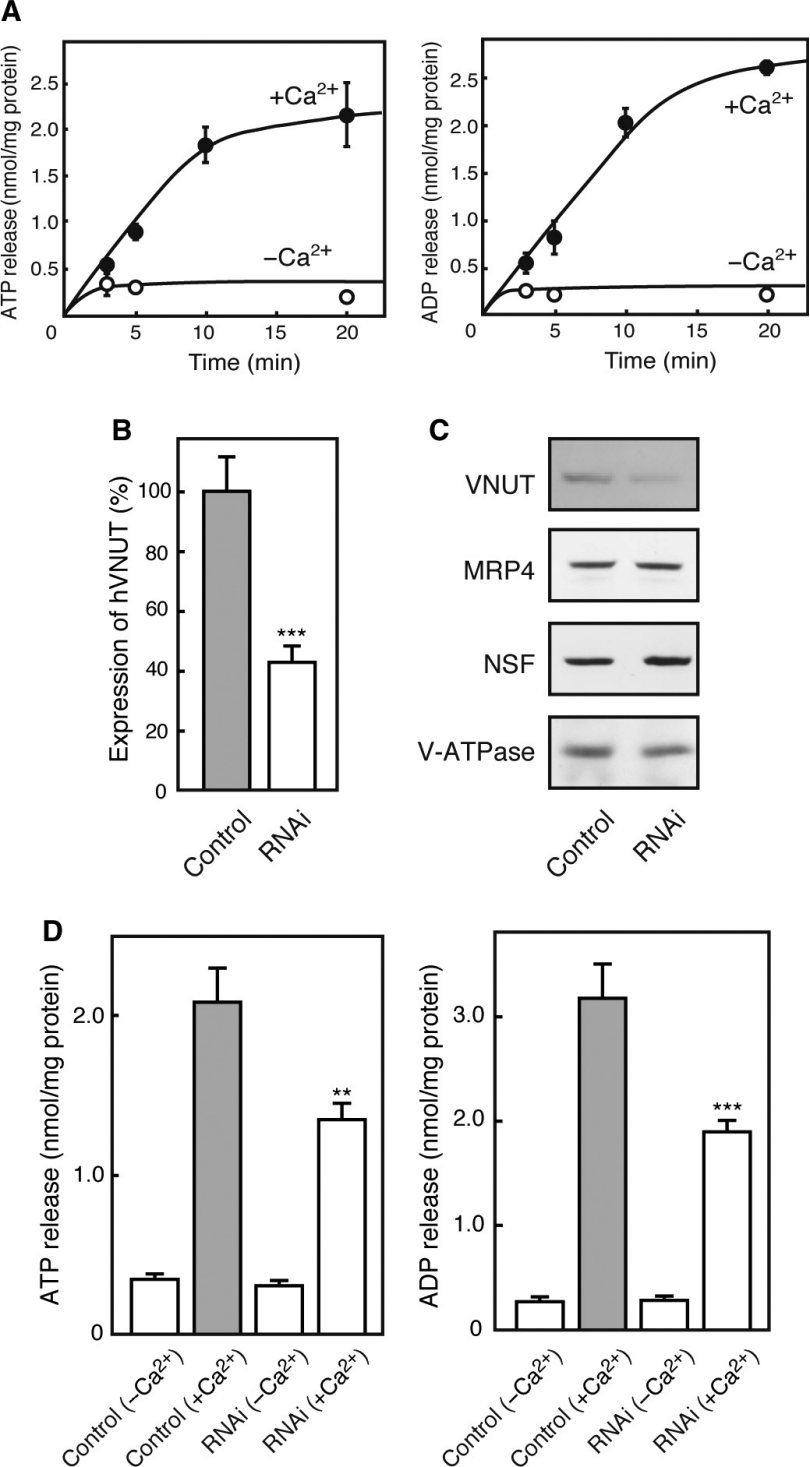
Vesicular nucleotide transporter (VNUT) was responsible for release of nucleotides from MEG‐01 cells. (A) Time course of ATP and ADP release in a Ca^2+^‐dependent manner from MEG‐01 cells. ATP release was initiated by the calcium ionophore A23187 at 5 *μ*mol/L. (B) RNAi against human VNUT decreased VNUT expression. Quantitative analysis for VNUT mRNA levels was performed by real‐time PCR. The amount of hVNUT mRNA was quantified as the ratio to G3PDH and expressed as relative to that of negative control siRNA. (C) Immunoblotting indicated that the concentration of VNUT protein decreases without affecting the expression of other proteins by RNAi. (D) Exocytosis of ATP and ADP were measured after 20 min the calcium ionophore A23187 stimulation at 5 *μ*mol/L from control siRNA‐treated MEG‐01 cells and VNUT siRNA‐treated MEG‐01 cells.

Then, we investigated whether VNUT gene expression is linked with the secretion of nucleotides from MEG‐01 cells. Nucleotide secretion was triggered by the addition of A23187, a Ca^2+^ ionophore, since it causes entry of extracellular Ca^2+^ into the cell interior; the resultant rapid increase in Ca^2+^ facilitates exocytosis of secretory vesicles. As shown in Figure 4A, appreciable amounts of ATP and ADP were released from the cells in a time‐dependent fashion. In the absence of extracellular Ca^2+^ in the medium, no A23187‐dependent release of nucleotides was observed.

The expression of the VNUT gene in MEG‐01 cells through RNA interference and the changes in nucleotide secretion were examined. RNA interference with a specific probe for VNUT decreased VNUT mRNA expression to ~40% of the control RNA (Fig. 4B). In agreement with this, the VNUT protein concentration decreased as revealed by immunoblotting, whereas the expression of other proteins such as MRP4, NSF, and subunit A of V‐ATPase was not affected (Fig. 4C). Under these conditions, A23187‐evoked ATP secretion from the siRNA‐treated cells was 60% of that of the control cells (Fig. 4D, left). A23187‐mediated ADP secretion was 60% of that of the control cells (Fig. 4D, right). Contents of ATP and ADP in control cells were 139.9 ± 2.4 and 32.1 ± 2.5 nmol/mg protein, respectively. Contents of ATP and ADP in siRNA‐treated cells were 142.3 ± 1.8 and 33.9 ± 3.7 nmol/mg protein, respectively, indicating that siRNA treatment had no effect on ATP/ADP content in MEG‐01 cells. Collectively, these results suggested that VNUT gene expression correlated well with Ca^2+^‐dependent secretion of nucleotides from MEG‐01 cells.

### Glyoxylate is a selective inhibitor of VNUT and suppressed both ATP uptake and release

**Figure 5.**
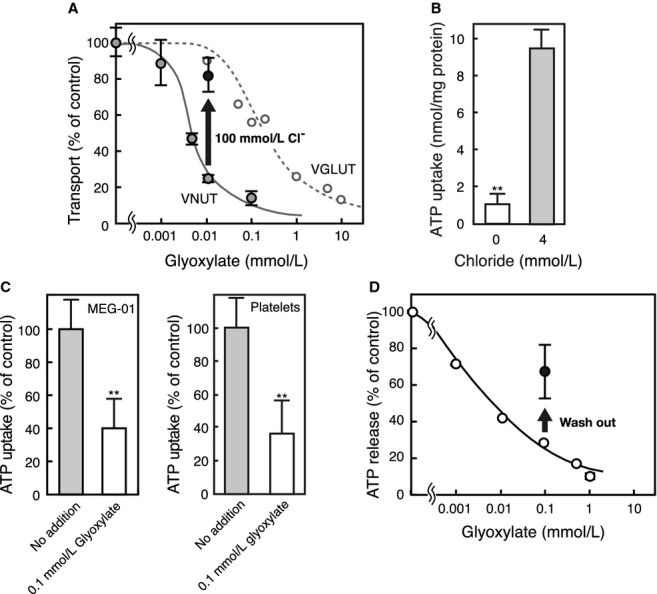
Glyoxylate is a potent and selective inhibitor for vesicular nucleotide transporter (VNUT). (A) The effect of glyoxylate on purified VNUT. The proteoliposomes containing purified human VNUT were incubated in buffer containing 20 mmol/L MOPS‐Tris (pH 7.0), 0.14 mol/L potassium acetate, 5 mmol/L magnesium acetate, and 10 mmol/L KCl. The effect of glyoxylate on glutamate transport by human VGLUT2 is also shown for comparison. (B) Cl^−^ dependence of ATP uptake by the membrane vesicles from MEG‐01 cells. The uptake was measured after 5 min in the presence or absence of Cl^−^. An equivalent concentration of the potassium acetate in the reaction mixture was replaced with the indicated concentration of KCl. (C) The effect of glyoxylate on ATP uptake by membrane vesicles of MEG‐01 cells (left) and platelets (right). ATP uptake was measured after 5 min in the absence or presence of 0.1 mmol/L glyoxylate. (D) Dose dependence of ATP release initiated by A23187 at 5 *μ*mol/L on glyoxylate concentration in MEG‐01 cells.

Finally, we confirmed the involvement of VNUT in vesicular storage and secretion of nucleotides from MEG‐01 cells by another line of experiment. It is known that ketone bodies modulate the Cl^−^ dependence of vesicular glutamate transporter (VGLUT) and reversibly suppresses glutamate exocytosis from glutamatergic neurons (Juge et al. 2010). This Cl^−^ dependence and reversible inhibition by ketone bodies are common properties of SLC17 members including VNUT (Juge et al. 2010). We looked for a more specific inhibitor of VNUT and found that glyoxylate inhibits VNUT reversibly and more preferentially than VGLUT (Fig. 5A). Concentration of glyoxylate required for 50% inhibition of VGLUT2 was 620 *μ*mol/L while that for VNUT was 4.1 *μ*mol/L (Fig. 5A, Table 2). Glyoxylate also inhibited vesicular excitatory amino acid transporter, VEAT, the fifth member of SLC17 family, to extents similar to that of VGLUT2 but was not inhibitory to vesicular monoamine transporter (VMAT), vesicular GABA transporter (VGAT), and vesicular acetylcholine transporter (VAChT) (Table 2). Subsequently, the effect of glyoxylate on ATP uptake by MEG‐01 membrane vesicles and ATP release by MEG‐01 cells was investigated. As expected, ATP uptake by the membrane vesicles required Cl^−^ (4 mmol/L) (Fig. 5B). Glyoxylate inhibited Cl^−^‐dependent ATP uptake in MEG‐01 and platelet membrane vesicles (40.6% and 36.6%, respectively) to approximately the same extent as that of purified human VNUT (cf Fig. 5A and C). Furthermore, glyoxylate effectively inhibited A23187‐evoked ATP release from MEG‐01 cells. This inhibition was partially recovered by removal of the substances by washing, which is consistent with the mode of inhibition of VNUT (Fig. 5D).

**Table 2.** Inhibitory potency of glyoxylate and acetoacetate toward vesicular neurotransmitter transporters

Transporters	IC_50_ (*μ*mol/L)	
	Glyoxylate	Acetoacetate
VNUT	4.1	190
VGLUT2	620	200
VEAT	52	130
VMAT2	N.I.	N.I.
VGAT	N.I.	N.I.
VAChT	N.I.	N.I.

Inhibitory potency of glyoxylate and acetoacetate toward various neurotransmitter transporters in 10 mmol/L Cl^−^ was assayed and shown as the concentration required for 50% inhibition. N.I., no inhibition observed by 10 mmol/L. VNUT, vesicular nucleotide transporter.

## Discussion

How nucleotides are stored in platelets is a long‐standing question. In the present study, we have conducted a series of experiments to examine the possible participation of VNUT in the storage process using human platelets and a line of human megakaryoblastic cells. We found that VNUT is localized in the intracellular granules containing serotonin. As serotonin is highly concentrated in the dense granule, the result indicates localization of VNUT in the dense granules of platelet (Da Prada et al. 1982).

Platelet membrane vesicles took up ATP in bafilomycin A_1_, Evans blue, and glyoxylate‐sensitive manners. Such kinetic features and substrate specificity are similar to those of purified VNUT. Background of minor ATP binding to membrane preparation and observed inhibitor profile indicates more than 50% of ATP uptake is estimated as VNUT‐driven transport.

In addition to histochemical and biochemical evidence, we also showed that the release of nucleotides from MEG‐01 cells was decreased by inhibition of VNUT activity and suppression of VNUT gene expression. Apparent molecular weight of VNUT immunological counterpart of platelet (~80 kDa) was higher than that of MEG‐01 counterpart (62 kDa). However, the migration position of platelet VNUT was shifted to ~55 kDa upon mild heat treatment in the presence of SDS. Therefore, it is possible that platelet VNUT may be tightly associated and complexed with other proteins in the platelet membrane so as to form a complex in vivo. Upon mild heat treatment, the complex may be dissociated and VNUT protein is electrophoresed to the standard position. The effect of *N*‐glycosidase F suggests that platelet VNUT is glycosylated. Collectively, all of these results constitute evidence that VNUT plays a central role in nucleotide accumulation in dense granules.

**Figure 6.**
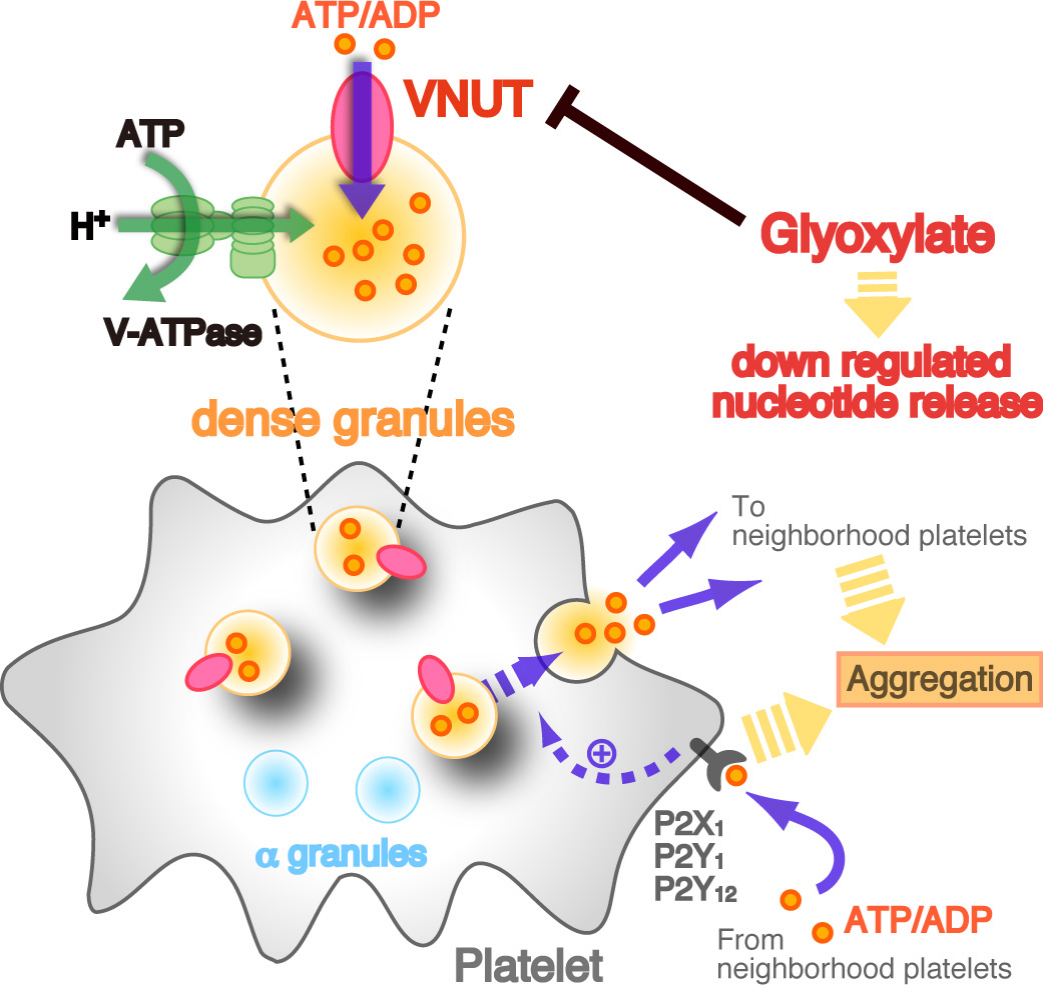
Schematic diagram of nucleotides storage and release in platelets. Vesicular nucleotide transporter (VNUT) is associated with dense granules and transports nucleotides into granule using *Δψ* that is established by V‐ATPase. VNUT is thus involved in nucleotide release and that is inhibited by glyoxylate, a VNUT inhibitor.

Biochemical studies on isolated dense granules suggest that V‐ATPase acts as a primary proton pump for the generation of an inside positive electrochemical gradient of H^+^ (*Δμ*H^+^, a sum of *Δ*pH and *Δψ*), which is used for serotonin uptake (Johnson et al. 1978; Carty et al. 1981; Wilkins and Salganicoff 1981; Fishkes and Rundnick 1982). Likewise, ATP uptake through VNUT in platelets seems to be coupled to V‐ATPase and uses *Δμ*H^+^ for the uptake of nucleotides. Thus, platelet dense granules may possess molecular machineries for the accumulation of transmitters that are essentially the same as those in the secretory vesicles of neurons and neuroendocrine cells (Fig. 6). The *Δ*pH and *Δψ* of platelet dense granules were evaluated with radiolabeled methylamine and through thiocyanate distribution and is estimated to be 1.1–1.6 and 30–40 mV, respectively (Johnson et al. 1978; Wilkins and Salganicoff 1981; Fishkes and Rundnick 1982). Since VMAT transports serotonin with a counterflow of 2H^+^ (Wilkins and Salganicoff 1981), this electrochemical gradient accounts for a 500‐ to 7000‐fold concentration gradient of serotonin across the granule membrane and, therefore, is sufficient for the storage of serotonin in the dense granules. As to nucleotide transport, VNUT utilizes *Δψ* to transport negatively charged ATP (Sawada et al. 2008). Under a *Δψ* of 40 mV, the ATP anion can be concentrated ~100‐fold, providing an explanation how intragranular ATP concentrations can reach ~700 mmol/L from a bioenergetics view (Omote et al. 2011; Omote and Moriyama 2013). In any event, the model presented here for the energy coupling of serotonin and nucleotides in dense granules explains quantitatively the vesicular storage of these compounds (Fig. 6).

MRP4 is another candidate for the transporter involved in the accumulation of ADP in dense granule (Jedlitschky et al. 2004, 2012). MRP4‐driven ADP transport may be hampered by ADP production through hydrolysis of its own ATP hydrolysis. In MEG‐01 cells, VNUT is expressed in MRP4‐ and cellubrevin‐positive dense granules and inhibition of VNUT expression by RNAi treatment reduced ATP release, whereas MRP4 protein expression level was not affected (Fig. 4C and D). In addition, kinetic properties of ATP uptake such as inhibitor sensitivity and Cl^−^ dependence are identical to those of VNUT (Figs. 3, 5B). Thus, MRP4 expression is not correlated with vesicular ATP uptake. These results suggested that although MRP4‐driven ADP transport may occur under physiological conditions, its physiological relevance for vesicular nucleotide storage is less significant.

One of the important findings of this study is that glyoxylate acted preferentially on VNUT and not on VGLUT. Recently we have shown that VGLUTs possess anion binding site(s) and that upon the binding of Cl^−^, VGLUTs become fully activated. Ketone bodies such as acetoacetate at mmol/L concentrations compete with Cl^−^ and hence, reversibly inhibit transport activity (Juge et al. 2010). Since acetoacetate‐evoked reversible inhibition of VGLUT occurs in vivo and the level of acetoacetate can be metabolically controlled, glutamatergic neurotransmission can be artificially and reversibly suppressed (Juge et al. 2010). VNUT is also activated by Cl^−^ and the activation can be reversibly inhibited by acetoacetate. This suggests the presence of a similar anion binding site(s) on VNUT and that vesicular ATP secretion may be regulated metabolically. The effect of glyoxylate on the vesicular transport as well as secretion fully supports this possibility.

One of the unique features of platelet dense granules is the presence of a high concentration of ADP (Holmsen 1958; Da Prada et al. 1982; Njus et al. 1986). Over 50% of the nucleotides in dense granules are ADP. Other secretory granules contain 10% ADP at the most (Zimmermann 1994). Taking the energy coupling in the dense granule presented here into consideration, the presence of a high concentration of ADP in dense granule is unexpected because VNUT almost equally recognizes ATP and ADP as substrates and the concentration of ADP in the cytoplasm is far less than that of ATP (Holmsen 1958). Thus, how ADP accumulates in the dense granules is an open question. One of the possibilities is that ADP is formed within the granules after storage; in other words, ATP is transported in through VNUT and is then hydrolyzed to ADP. In this respect, it is noteworthy that dense granules also contain relatively high concentration of polyphosphate, which is synthesized inside granules and released along with ADP and serotonin and also acts as a novel transmitter to facilitate clotting (Smith et al. 2006; Müller et al. 2009). The enzymes responsible for the synthesis of polyphosphate in platelets are unknown at present. We speculate that the synthesis of polyphosphate and formation of ADP inside granules is linked. It is noteworthy that MEG‐01 cells secrete appreciable amount of ATP and ADP in a Ca^2+^‐dependent manner (Fig. 4). Suppression of VNUT gene expression equally suppressed secretion of ATP and ADP without affecting cytoplasmic ATP/ADP ratios (Fig. 4). These results strongly suggest that MEG‐01 cells also store high concentration of ADP in the vesicles and intragranular ADP is somehow endogenously produced. Although it is unknown whether MEG‐01 cells contain appreciable amount of polyphosphate, the cells may provide suitable experimental system to analyze molecular mechanism by which platelets store and secrete high concentration of ADP. Further studies in particular identification of polyphosphate in MEG‐01 cells and, if present, coupling of formation of ADP and polyphosphate will be necessary.

In conclusion, we demonstrated that VNUT played an essential role in vesicular nucleotide transport and release in platelets. Identification of glyoxylate as a reversible inhibitor of VNUT provides a lead for the development of selective modulators of VNUT activity to control nucleotides release in vivo.

## Acknowledgments

We wish to thank R. Taguchi, Y. Kato, S. Moriyama, and Y. Sugimoto at Okayama University for the help throughout the study. We also thank T. Nishi at ISIR of Osaka University for providing MEG‐01 cells.

## Conflict of Interest

None declared.
